# Advances in Targeted Therapies for Head and Neck Cancer Using Polymer-Based Nanoparticles

**DOI:** 10.34172/apb.45968

**Published:** 2026-05-24

**Authors:** Mina Afrashteh Nour, Maryam Kouhsoltani, Hamed Hamishehkar

**Affiliations:** ^1^Department of Biochemistry, Faculty of Medicine, Tabriz University of Medical Sciences, Tabriz, Iran; ^2^Biotechnology Research Center, Tabriz University of Medical Sciences, Tabriz, Iran; ^3^Department of Oral and Maxillofacial Pathology, Faculty of Dentistry, Tabriz University of Medical Sciences, Tabriz, Iran; ^4^Drug Applied Research Center, Tabriz University of Medical Sciences, Tabriz, Iran; ^5^New Material and Green Chemistry Research Center, Khazar University, 41 Mehseti Street, AZ1096, Baku, Azerbaijan

**Keywords:** Head and neck cancer, Oral squamous cell carcinoma, Polycaprolactone, Polymer-based nanoparticles, Poly(lactide-co-glycolide), Targeted therapy

## Abstract

Head and neck cancer (HNC), the seventh most common cancer type, remains associated with high mortality rates despite the use of current treatment modalities such as surgery, radiotherapy, and chemotherapy. Owing to the side effects and lack of specificity of these standard treatments, polymeric nanoparticles (PNPs) have emerged as promising cancer therapeutics by enabling improved drug delivery to neoplastic sites and reducing off-target toxicity. This review summarizes advances in PNP-based therapies for HNC, with a particular focus on major systems such as polycaprolactone (PCL), poly(lactide-co-glycolide) (PLGA), chitosan, and polyethyleneimine nanoparticles, as applied in chemotherapy, gene therapy, and combination phototherapies. In oral squamous cell carcinoma (OSCC), PCL copolymers have been shown to induce apoptosis, inhibit tumor growth, and enable dual drug delivery and photothermal/photodynamic therapy. PLGA formulations enhance the efficacy of chemotherapeutics such as paclitaxel and gemcitabine/cisplatin in pharyngeal and nasopharyngeal cancers through controlled and sustained release. Chitosan-based systems have been reported to provide mucoadhesion, improved residence time at mucosal sites, and efficient gene silencing. Overall, these advances highlight the potential of rationally engineered PNPs to address current therapeutic limitations in HNC. However, further work is required to optimize PNP design, fully characterize long-term toxicity, and validate clinical benefit in rigorously designed clinical trials.

## Introduction

 Head and neck cancer (HNC), which accounts for nearly 325,000 deaths annually, encompasses a diverse group of epithelial malignancies arising within the upper aerodigestive tract, including the paranasal sinuses, nasal cavity, oral cavity, pharynx, and larynx.^1^ Exposure to tobacco-derived carcinogens, excessive alcohol consumption, or both, substantially contributes to the burden of these heterogeneous malignancies. Early detection of HNC is challenging and relies partly on physical examination because of the absence of practical population-level screening tests. The Staging differs according to the anatomic site and ranges from small, localized primary tumors to advanced disease with extensive lymph node involvement. Multiple therapeutic modalities are available for HNC and are frequently used in combination. These include radiotherapy, surgery, and chemotherapy, selected according to tumor type, primary site, and disease stage.^2^ Despite such multimodal approaches, 5-year survival remains approximately 50–60%, limited by chemoresistance—for example, drug efflux via ATP-binding cassette (ABC) transporters—and poor drug penetration into the tumor microenvironment.^3,4^ Certain subtypes of head and neck squamous cell carcinoma (HNSCC), such as HPV-positive oropharyngeal squamous cell carcinoma (OPSCC), exhibit greater radiosensitivity; however, recurrence rates in advanced stages still exceed 40%.^3,5^ Recent advances in molecular oncology have prompted the exploration of nanotechnology as a complementary strategy to conventional therapies in HNC. Nanoparticle (NP) drug delivery systems exploit nanoscale materials to improve pharmacokinetics, increase tumor accumulation, and reduce systemic toxicity. Their distinctive features, including precise targeting and the possibility of stimulus-responsive drug release, are particularly attractive in the head and neck region with its complex and delicate anatomy. Future treatment of HNC is likely to be shaped by personalized medicine and immunotherapy, providing patients with the prospect of improved outcomes and quality of life.^6,7^ A wide range of “smart” NPs has now been developed that respond to internal or external stimuli; among the most prominent are polymeric nanoparticles, dendrimers, and micelles, as well as biomimetic systems such as liposomes, protein NPs, and cell membrane-coated NPs, and various inorganic platforms.^8^

 This review focuses on polymer-based nanoparticles in targeted therapy for HNC, describing their forms, compositions, and pharmaceutical applications. It also highlights preclinical and early translational data in oral, pharyngeal, laryngeal, and nasopharyngeal malignancies.

## Pathogenesis and Treatment Landscape of Head and Neck Cancer

 HNCs rank as the seventh most prevalent malignancy worldwide and comprise a heterogeneous group of tumors arising from various anatomical sites within the head and neck region, each with distinct clinical and biological features. Increasing evidence indicates that this heterogeneity contributes to high mortality rates and treatment failures, particularly with conventional therapies.^9^ HNC accounts for roughly 3% of all malignancies in the United States each year, with a recent rise mainly attributable to an increase in oropharyngeal squamous cell carcinoma (OPSCC).^10^ Approximately 90% of HNCs are head and neck squamous cell carcinoma (HNSCC), originating from the mucosal epithelium of the oral cavity, pharynx, and larynx. Major risk factors include cigarette smoking, alcohol consumption, and infection with high-risk human papillomavirus (HPV), particularly types 16 and 18. Despite the introduction of prophylactic HPV vaccination, a substantial proportion of cases remain HPV-negative HNSCC.^11^

 Epstein–Barr virus (EBV) infection is a recognized risk factor for nasopharyngeal carcinoma (NPC).^12^ Other contributors, including alterations in the microbiota and dietary patterns, have also been linked to HNSCC in recent decades.^13^ Early diagnosis is only partially achievable via physical examination in the absence of effective screening tools, and staging varies by anatomical location, from small primary tumors to advanced disease with regional lymph node metastasis.^14,15^ In laryngeal cancer, 5-year disease-free survival rates in stage I and II disease are approximately 90% and 80%, respectively.^16^

 HNSCC is driven by genetic and epigenetic alterations that affect key biological processes such as cell proliferation, cell-cycle regulation, apoptosis, and metastasis. These include gain-of-function mutations in oncogenes and loss-of-function mutations in tumor suppressor genes, as summarized schematically in Figure 1.^17-25^

**Figure 1:**
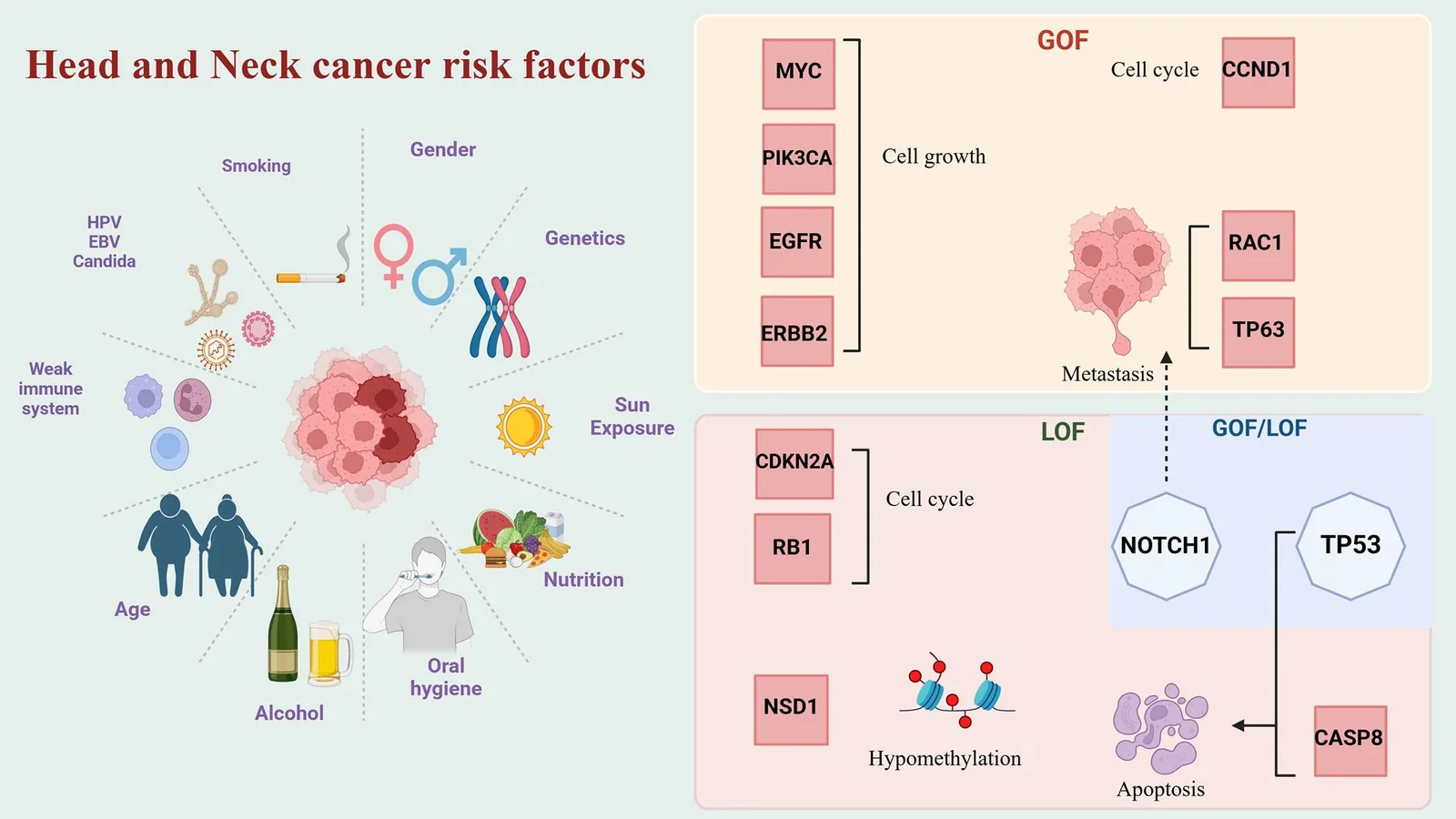
Head and neck cancer risk factors, gain-of-function (GOF) and loss-of-function (LOF) mutations, and related gene regulation in head and neck cancer (created with BioRender)

 Treatment options for HNC vary according to tumor type, primary site, and stage. Radiotherapy or surgery is typically recommended for early-stage disease. In contrast, chemotherapy is often combined with radiotherapy or surgery in more advanced stages and is also used in head and neck lymphomas.^26^ In addition, small interfering RNA (siRNA)-based inhibition of key signaling molecules involved in HNSCC pathogenesis, such as Akt1 and Jak1, has emerged as a targeted strategy to enhance chemosensitivity.^27^

 In oral cavity cancers, surgery is often followed by adjuvant radiotherapy or chemotherapy, whereas cancers of the throat and larynx are more frequently treated with chemoradiotherapy. Ongoing research suggests that de-escalation of treatment intensity may be appropriate for selected HPV-positive HNSCC patients.^28^ Immune checkpoint inhibitors such as nivolumab and pembrolizumab have been approved by the U.S. Food and Drug Administration (FDA) for metastatic or recurrent HNSCC, representing a major advance in systemic therapy.

 With recent developments in nanotechnology, particularly polymeric nanoparticles (PNPs), several limitations of current treatments are being addressed through enhanced permeability and retention (EPR) effects, ligand-mediated targeting, and controlled drug release within the acidic and hypoxic tumor microenvironment characteristic of HNC. Detailed discussions of NP-based drug delivery systems are provided in the following section.

## Nanoparticle-Based Drug Delivery Systems

 According to the International Organization for Standardization (ISO), nanoparticles (NPs) are nano-objects with dimensions on the nanoscale whose longest and shortest axes do not differ appreciably. Nanomedicine drug delivery systems employ nanoscale materials to direct pharmaceuticals to specific sites in the body, thereby improving therapeutic efficacy in chronic diseases and cancers.^29^ NPs typically range from 1 to 100 nm in size and can adopt a variety of shapes, including spherical, conical, and cylindrical morphologies. They may be composed of single or multiple layers, often comprising distinct core, shell, and surface domains with different chemical characteristics.^30,31^ NPs are commonly classified into three broad categories according to their composition: organic (such as micelles, liposomes, and dendrimers), carbon-based, and inorganic (such as magnetic, semiconductor, lanthanide, and metallic NPs) (Figure 2).^32^ Organic nanoparticles (NPs) are preferred for targeted drug delivery because of their biodegradability and low toxicity.^31^ Carbon-based NPs, composed entirely of carbon atoms—such as fullerenes, carbon black nanoparticles, and carbon quantum dots—are well known for their unique physicochemical properties in drug delivery, bioimaging, and energy storage.^33,34^ NPs, including metallic, ceramic, and semiconductor nanoparticles, possess distinctive properties such as specific optical and electrical characteristics in metallic particles and high structural stability in ceramic forms. These features make them valuable in a wide range of applications, from biomedical engineering to optoelectronics.^33,35^

**Figure 2:**
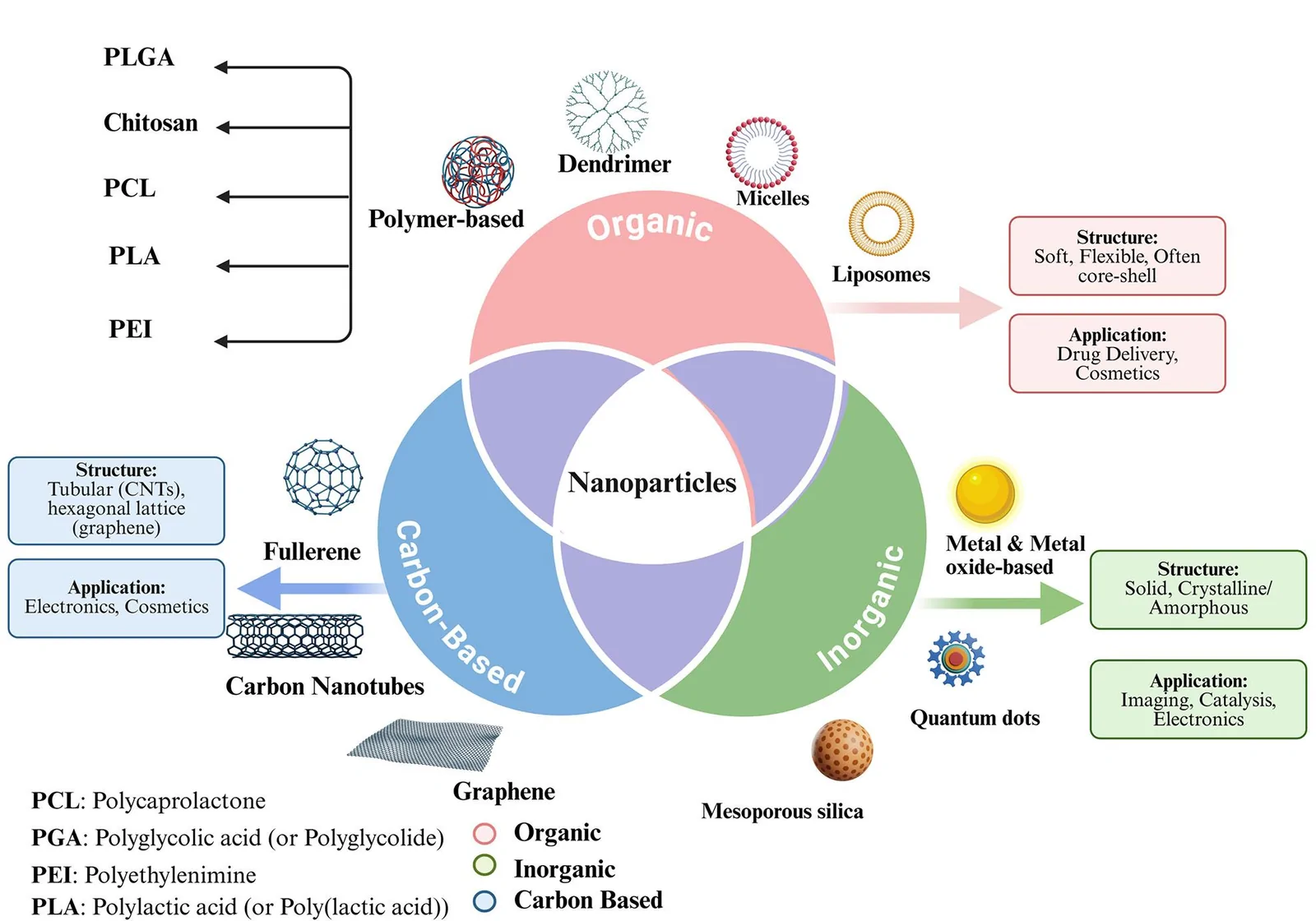
Classification of Nanoparticles. Nanoparticles can be classified into three main classes including inorganic, organic,and carbon-based. Lipid-based organic nanoparticles are also included among the types of organic nanoparticles as well, while polymer-based nanoparticles refer to common polymers of PLGA, PCL-PEG, PEI, PLA and chitosan (Created with bio render)

 Nanoparticles can function through either active or passive mechanisms, achieved by direct conjugation of therapeutic agents to the carrier surface or by encapsulation within the nanoparticle structure. Because nanoparticle performance strongly depends on structural parameters such as size, morphology, and surface characteristics, significant research efforts focus on optimizing these features to improve targeted delivery while minimizing toxicity.^36^ Several analytical techniques are used to characterize nanoparticles. Scanning electron microscopy (SEM), transmission electron microscopy (TEM), and scanning tunneling microscopy (STM) are widely applied for structural analysis. SEM and TEM enable evaluation of particle size, aggregation behavior, and crystal structure through electron beam imaging, whereas STM analyzes surface topography using quantum tunneling principles.^37^ In addition, X-ray diffraction (XRD) is frequently employed to determine nanoparticle phase composition and crystallinity through analysis of incident and scattered X-rays.

 Nanoparticles possess unique physicochemical properties that offer the potential to revolutionize cancer treatment. For instance, metal-based nanoparticles such as gold (Au) and gadolinium (Gd) can enhance tumor-specific magnetic resonance imaging (MRI) contrast and support selective photothermal therapy.^38^

 Furthermore, nanoparticles such as ZnO and Fe₃O₄ have demonstrated promising capabilities in targeted drug delivery and tumor cell destruction.^39^ Although nanoparticle-based drug delivery systems can significantly improve therapeutic targeting and potency, challenges such as potential toxicity and immune clearance remain. Ongoing research therefore focuses on engineering multifunctional nanoparticles with optimized physicochemical characteristics to overcome drug resistance and support biomarker-guided immunotherapy and personalized treatment strategies. The following section discusses the importance of polymeric nanoparticles in drug delivery.

## Polymeric Nanoparticles for Drug Delivery

 Polymeric nanoparticles (PNPs) are solid matrix systems in which pharmaceutical compounds are either encapsulated within or attached to polymer scaffolds. These systems can carry both macromolecules, such as proteins and nucleic acids, and small hydrophilic or hydrophobic drugs. Their physicochemical characteristics—including size, shape, molar mass, zeta potential, and stability—directly influence their therapeutic performance.^40,41^ PNPs generally range from 1 to 1000 nm in diameter and can be engineered to target specific therapeutic applications.^42,43^ For example, tumor vasculature can accommodate nanoparticles approximately 100 nm in size, whereas smaller particles (~70 nm) may demonstrate improved penetration into brain tumors. However, excessively small nanoparticles may be rapidly cleared or neutralized by the immune system, highlighting the need for careful optimization of particle size.^44^ Overall, parameters such as particle size, morphology, polymer composition, surface charge, and biological stability play critical roles in determining therapeutic efficacy.

 Common polymeric scaffolds include FDA-approved natural polymers such as chitosan and albumin, as well as synthetic polymers including poly(lactide) and poly(lactide-co-glycolide) (PLGA). Synthetic polymers are increasingly favored because of their tunable physicochemical properties and cost-effectiveness.^45^ Among various nanoparticle morphologies, spherical PNPs are particularly advantageous because they often exhibit improved encapsulation efficiency, stability, and pharmacokinetic performance. Their targeting ability can be further enhanced through conjugation with ligands such as folic acid or antibodies that recognize specific receptors on cancer cell surfaces.^43^

 PNPs can prolong drug half-life and enable controlled drug release by facilitating targeted accumulation within tumor tissues. As a result, higher effective drug concentrations can be achieved at resistant tumor sites while minimizing systemic exposure. This targeted delivery significantly reduces off-target toxicity, which is one of the major limitations of conventional chemotherapy.^46^ Engineered PNPs are currently being explored for the treatment of multiple malignancies, including colorectal, breast, and pancreatic cancers. In addition, their capacity to cross the blood–brain barrier provides promising opportunities for the treatment of brain tumors and neurological disorders. In the context of HNC, similar nanoparticle design principles are increasingly applied to improve local drug delivery and reduce systemic adverse effects.

 Among the polymers used in nanoparticle design, polyethylene glycol (PEG) plays an important role in improving nanoparticle performance. PEG is a linear polymer containing reactive hydroxyl groups that can modify nanoparticle surfaces. PEGylation increases nanoparticle hydrophilicity, improves pharmacokinetics, reduces toxicity, and minimizes interactions with plasma proteins during systemic circulation.^47^

 Block copolymers have also emerged as important components in targeted drug delivery systems due to their ability to self-assemble into nanoparticles, micelles, and hydrogels. Polymeric nanoparticles derived from block copolymers—such as mPEG-PLA, PCL-PEG, and PEG-PEI—are widely applied in cancer therapy, with each polymer system offering distinct advantages for drug delivery.^48^ To achieve optimal lipophilicity, surface charge, and biocompatibility for specific therapeutic applications, various nanoparticle synthesis techniques are employed. These include nanoprecipitation, emulsion-diffusion, emulsification-coacervation, double emulsification, surface polymerization, and layer-by-layer assembly. In addition, advanced polymerization methods such as atom transfer radical polymerization (ATRP) allow precise fabrication of multifunctional polymer structures tailored for specific therapeutic needs.^48,49^

 An important advancement in this field is the development of stimuli-responsive polymers that respond to environmental triggers such as temperature, pH, light, oxidative conditions, or redox potential. These smart polymer systems enable controlled and site-specific drug release, providing a promising strategy for precision cancer therapy.

 Overall, by adjusting polymer composition, architecture, and responsiveness, polymeric nanoparticles provide versatile platforms capable of overcoming pharmacokinetic and biological barriers that are particularly relevant in head and neck malignancies.

## Toxicity and Biodistribution Studies

 Nanotechnology-based drug delivery platforms have significantly improved the treatment of HNCs by enhancing tumor biodistribution and reducing systemic toxicity compared with conventional free drugs.^50^ Traditional chemotherapeutic agents often suffer from poor biodistribution, rapid clearance, and non-specific tissue accumulation, leading to severe adverse effects such as mucositis, renal toxicity, and hepatotoxicity in patients with HNSCC.^51^

 Inorganic nanoparticles may accumulate uncontrollably in organs such as the liver and spleen, potentially causing toxicity. In contrast, polymeric nanoparticles demonstrate more favorable biodistribution profiles, lower toxicity, and the capacity to encapsulate a broad range of therapeutic agents.^50^ Owing to the enhanced permeability and retention (EPR) effect, these nanoparticles tend to accumulate preferentially at tumor sites while maintaining prolonged circulation times.^52^

 For example, Badiee et al. (2022) reported that nano-formulated anti-PD-1 antibodies showed significantly reduced distribution in off-target tissues while maintaining strong accumulation in tumors and lymph nodes.^53^ Another study published in 2025 demonstrated that engineering polymeric nanoparticles within the size range of 10–1000 nm can substantially reduce systemic toxicity by improving biocompatibility and enabling sustained drug release.^52^ In murine HNSCC models, paclitaxel-loaded polymeric nanoparticles (PTX-NPs) significantly reduced systemic toxicity compared with free paclitaxel, including reduced bone marrow suppression and neurotoxicity. Improved pharmacokinetic profiles were attributed to higher tumor concentrations and reduced off-target uptake. In these studies, PTX-NPs exhibited lower toxicity even at doses of 5 mg/kg.^54^

 Additionally, PLGA-PVA nanoparticle platforms have been developed to enable localized multidrug delivery, imaging, and fiducial marker placement for radiotherapy targeting in HNC. Bi-layered polymer systems composed of calcium carbonate and thymoquinone have demonstrated accurate tumor localization, favorable biodistribution, and minimal toxicity in xenograft mouse models. These systems also support simultaneous delivery of immunotherapy and chemotherapy agents.^55^

 Biocompatibility evaluations by Pellionisz et al. showed no significant inflammation or toxicity in histopathological analyses following radiotherapy combined with polymer-based cisplatin formulations.^56^ These findings support the potential of polymeric nanoformulations as safer drug delivery systems. However, certain limitations remain. Acidic degradation products from polymer breakdown may cause local irritation in some cases, and PEGylation may trigger rare immune responses, such as anti-PEG antibody formation. Therefore, careful monitoring is required in clinical applications.^57,58^

 Despite promising preclinical findings, further evidence from well-designed clinical trials is still needed.^59^

## Clinical Translation

 Although nanoparticle-based therapies have demonstrated considerable promise in preclinical studies, their translation into clinical practice remains challenging. Lima et al. analyzed 1,747 studies and found that only eight met the criteria for meta-analysis, indicating that relatively limited clinical progress has been achieved and that most studies have not yet translated into clear clinical benefits for patients with HNC.^59^ Several factors contribute to the slow clinical progress, including the complex and immunosuppressive tumor microenvironment as well as high levels of drug resistance.^60^ Nevertheless, polymeric nanoparticles continue to show strong potential for clinical translation in HNC therapy. These systems—based on biodegradable or non-biodegradable polymers—can provide controlled drug release, extended circulation time, and scalable production.

 Surface modifications further improve pharmacokinetics and targeting capability by enabling selective recognition of tumor cells. Emerging platforms such as polymeric hydrogels are also being explored for biomedical applications. Collectively, these properties make polymeric nanoparticles promising tools for overcoming biological barriers in medical treatments.^50^

 Several clinical trials are currently evaluating nanoparticle-based therapies. A phase II clinical trial (NCT06366945) is investigating the use of tislelizumab (anti-PD-1) combined with carboplatin and polymeric micellar paclitaxel as neoadjuvant therapy for resectable HNSCC with lymph node metastases. The nanoparticle formulation aims to enhance tumor penetration, promote immunogenic cell death, improve induction therapy efficacy compared with solvent-based paclitaxel, and reduce distant metastases.

 Another clinical trial (NCT06301165) compares polymeric micellar paclitaxel within a TPC regimen (paclitaxel, cisplatin, and capecitabine) against a GP regimen (gemcitabine and cisplatin) for high-risk nasopharyngeal carcinoma, aiming to improve tumor penetration and retention.

 However, some earlier trials have shown limited progress. For example, the phase II study NCT02639858 investigating docetaxel-loaded polymeric micelles in recurrent or metastatic HNSCC has remained incomplete since 2017. Another study (NCT05456022) comparing free quercetin formulations with PLGA-PEG nanoparticle-encapsulated quercetin reported an unusually high enrollment figure, suggesting that it may represent a laboratory-based experimental study rather than a true clinical trial. Additionally, the phase I trial NCT02817113 evaluating NC-6004 (a polymeric micellar formulation of cisplatin) combined with 5-fluorouracil and cetuximab was terminated after enrolling only four patients due to strategic changes.

 Overall, clinical research on polymeric nanoparticles in HNSCC remains at an early stage. Most ongoing trials focus on micellar formulations designed to increase tumor permeability and reduce the toxicity associated with taxane- and platinum-based chemotherapies. Despite promising preclinical results, as of December 2025 no published clinical evidence has definitively confirmed their therapeutic efficacy in patients.

## Role of polymer-based nanoparticles in HNC Therapy

 Despite the significant mortality caused by HNC, there is hope in the potential of PNPs in cancer therapy. The American Cancer Society’s data, along with the advancements in diagnosis and therapy, have not been able to significantly reduce the side effects of the multitude of therapeutic interventions. However, early diagnosis remains a fundamental approach to enhance the disease prognosis and minimize the impact on other organs. The use of NPs of diverse sizes and shapes that can overcome biological barriers represents an innovative approach to cancer management.^61^ This section will delve into the promising application of various polymer NPs, independently or as copolymers, specifically in the context of HNCs.

###  Copolymers

####  PCL/ PCL Copolymers

 Poly(ε-caprolactone) (PCL) and its copolymers are being studied in HNC research due to the ability to carry diverse therapeutic payloads.^62^

 Across studies, amphiphilic copolymers such as PEG–PCL and its derivatives dominate, reflecting their biocompatibility and tunable degradation profiles, while hybrid or functionalized systems demonstrate superior gene or drug delivery efficiency.

 In vitro findings collectively indicate that polymer architecture and surface charge are decisive for cellular uptake and cytotoxic efficacy. Cationic micelles, such as DOTAP-mPEG-PCL, showed a high transfection efficiency (around 52%) and triggered apoptosis in SCC15 cells as a result of the electrostatic attraction between positively charged micelles and negatively charged cell membranes.^63^

 Furthermore, PEG-PCL micelles containing icaritin^64^ or curcumin^65^ demonstrated increased aqueous solubility, cellular uptake, dose-dependent toxicity, and reduced off-target toxicity compared to free drug forms. These findings indicate that hydrophobic PCL cores enhance loading and release control, while PEG shells prolong circulation and minimize immune recognition, balancing efficacy and safety.

 Functional modifications further improved selectivity. Redox-responsive PEG–PCL NPs with folate targeting achieved enhanced tumor accumulation in FA-receptor–positive OSCC via disulfide bond cleavage, yielding superior inhibition compared with free paclitaxel or nontargeted controls. In contrast, nonresponsive PEG–PCL carriers required higher doses for equivalent effects, highlighting the advantage of stimuli-sensitive linkages in redox-rich tumor environments.^66^

 Using near-infrared irradiation, PEG-PCL-C3-ICG NPs provided the capability of photothermal and photodynamic therapy in OSCC models with minimal systemic cytotoxicity. Organic PEG–PCL formulations exhibited faster metabolic clearance, lower off-target toxicity, and superior biocompatibility than metallic agents, suggesting the translational potential of biodegradable and metabolically stable polymeric nanocarriers for combined therapy.^67^

 Furthermore, chitosan-coated PCL NPs exhibited mucoadhesive and cationic properties, which enhanced mucosal retention and cellular uptake. The combination of chitosan and 5-fluorouracil enhanced antitumor efficacy in HNSCC models. However, immunogenicity and batch heterogeneity of chitosan, especially for repeated mucosal administration, remain translational challenges.^68^

 A work by Chen et al. similarly indicated that TPGS-PCL copolymers encapsulating HIF-1α siRNA achieved effective gene silencing and tumor suppression in nasopharyngeal cancer; however, long-term stability and large-scale repeatability require additional confirmation.^69^

 Moreover, a dual-drug PCL NP formulation containing cisplatin and curcumin demonstrated synergistic effects in inhibiting oral carcinoma cells, demonstrating the efficacy of NP combinations in overcoming drug resistance. Such designs highlight the potential of co-delivery systems to exploit multiple molecular pathways; however, variability in release kinetics and pharmacokinetic synchronization still limit translation.^70^

 PCL-Pluronic micelles have shown synergistic efficacy when combined with genetic adjuvants. In esophageal carcinoma models, Dai et al. showed that PCL-Pluronic micelles loaded with doxorubicin and miR-34a enhanced apoptosis and tumor suppression. Combined with miRNA, Pluronic segment enhanced membrane permeability and endosome escape, resulting in synergistic antitumor activity. The data supports the conclusion that dual chemo-gene strategies utilizing amphiphilic block copolymers can deliver higher therapeutic outcomes (Table 1).^71^

**Table 1:** Polycaprolactone (PCL)/ PCL copolymer NPs for HNC therapeutics

**Cancer Type**	**Polymer**	**Active Ingredient**	**Model Systems**	**Particle Characteristics**	**Dosing / Control Arms**	**Outcomes (IC**_50_ ** shift, tumor inhibition, etc.)**	**Short Analysis (Why some approaches performed better)**	**Ref.**
Oral squamous cell carcinoma (OSCC)	mPEG–PCL + DOTAP hybrid micelles (DMP)	BimS gene (phBimS plasmid)	*In vitro:* SCC15; *In vivo:* mouse tongue xenograft	28 nm; ζ = + 43 mV; spherical	Local & IV vs saline & blank DMP	52 % transfection; apoptosis 33.9 %; tumor inhibition 65.7 % (local), 45.6 % (systemic)	Lipid–polymer hybrid enhanced gene delivery via caveolin-mediated endocytosis and strong tumor retention.	^63^
OSCC	PEG–PCL encapsulating C3 + ICG	C3 (PTT) + ICG (PDT)	*In vitro* OSCC; *In vivo* OSCC mice	60–80 nm; spherical; photostable	Laser 808 nm vs ICG, AuNRs, PBS	ROS ↑; complete ablation; minimal toxicity	Dual PTT/PDT effect with biocompatible polymer–photosensitizer synergy improved precision and safety.	^67^
OSCC	PEG–PCL micelles	Icaritin	*In vitro:* OSCC cells	121 nm; spherical; sustained release	Free vs encapsulated	Enhanced uptake, cytotoxicity; improved solubility	PEG–PCL improved stability and intracellular delivery of hydrophobic flavonoids.	^64^
Oral cavity SCC	PCL NPs + chitosan coating	Curcumin	*In vitro:* SCC-9; *Ex vivo:* porcine mucosa	~200 nm; mucoadhesive; positive ζ	Free Cur vs uncoated vs coated NPs	↓ Cell viability; ↑ mucosal retention	Chitosan improved mucoadhesion and local drug residence, minimizing systemic exposure.	^65^
Oral epidermal carcinoma	PCL NPs (PVA + Tween 80)	Cisplatin + Curcumin	*In vitro:* Oral carcinoma cells	< 300 nm; 70 % Cur; 80 % CDDP loading	Free vs single vs dual-drug NPs	Synergistic cytotoxicity (CI < 1)	Dual-drug encapsulation enabled complementary cytotoxic pathways and reduced free-drug toxicity.	^70^
HNSCC	Chitosan–PCL hybrid microparticles	5-Fluorouracil (5-FU)	*In vitro:* CAL27, HSC3; *In vivo:* AT84 mouse	MPs; EE = 38.6 %; release ≤ 96 h	5-FU CS–PCL vs free 5-FU	Growth inhibition; ↑ LC3-II, PARP1 cleavage	CS coating enhanced retention and autophagy-linked apoptosis.	^68^
OSCC	FA–PEG–S–S–PCL (redox-responsive)	Paclitaxel (PTX)	*In vitro:* HSC3; *In vivo:* xenograft mice	90–120 nm; GSH-sensitive; disulfide bonds	Free PTX vs PEG–S–S–PCL@PTX vs FA–NP@PTX	↑ Tumor uptake; best inhibition	FA targeting + redox cleavage achieved selective intracellular PTX release.	^66^
Esophageal SCC	PCL–Pluronic micelles	Doxorubicin + miR-34a	*In vitro:* ESCC cells; *In vivo:* mouse xenograft	Nanosized; improved uptake vs free DOX	DOX-PCL ± miR-34a vs controls	Synergistic tumor suppression > single treatments	miR-34a restored chemosensitivity; PCL–Pluronic boosted drug accumulation.	^71^
Nasopharyngeal carcinoma	TPGS-b-(PCL-ran-PGA) diblock copolymer	siRNA targeting HIF-1α	*In vitro:* CNE-2; *In vivo:* xenograft mice	Biodegradable; ~150 nm; EE ≈ 80 %	siRNA-NP vs naked siRNA vs saline	↓ HIF-1α mRNA & protein; ↑ cell death; strong tumor suppression	Amphiphilic TPGS improved endosomal escape and siRNA stability; potent hypoxia-gene suppression.	^69^

Abbreviations: mTHPC: *meta*-Tetra(hydroxyphenyl)chlorin, OSCC: Oral squamous cell carcinoma, mPEG: methoxy poly(ethylene glycol), PCL: poly(ε-caprolactone), DOTAP: 1,2-dioleoyl-3-trimethylammonium-propane, DMP: DOTAP-mPEG-PCL hybrid micelles, ζ: zeta potential, IV: intravenous, PEG: poly(ethylene glycol), PTT: photothermal therapy, ICG: indocyanine green, PDT: photodynamic therapy, AuNRs: gold nanorods, PBS: phosphate buffered saline, ROS: reactive oxygen species, SCC: squamous cell carcinoma, NPs: nanoparticles, Cur: curcumin, PVA: polyvinyl alcohol, CDDP: cis-diamminedichloroplatinum(II), CI: combination index, HNSCC: head and neck squamous cell carcinoma, 5-FU: 5-Fluorouracil, MPs: microparticles, EE: encapsulation efficiency, LC3-II: microtubule-associated protein 1 light chain 3-II, PARP1: poly(ADP-ribose) polymerase 1, FA: folic acid, S–S: disulfide bond, PTX: paclitaxel, GSH: glutathione, ESCC: esophageal squamous cell carcinoma, DOX: doxorubicin, miR-34a: microRNA-34a, TPGS: d-α-tocopheryl polyethylene glycol succinate, PGA: poly(glycolic acid), siRNA: small interfering RNA, HIF-1α: hypoxia-inducible factor 1-alpha

 Generally, stimuli-responsive PNPs have shown improved therapeutic efficacy due to their improved cellular uptake and controlled drug release properties. However, their use in clinical conditions is hampered due to several biological barriers and the fact that their studies have not been standardized. Future work should emphasize standardized evaluation models and cross-polymer comparisons to determine which delivery strategies offer the most realistic translational potential for HNC therapy.

####  PLGA/ PLGA Copolymers

 Poly(lactide-co-glycolide) (PLGA) NPs, copolymer of poly lactic acid (PLA) and poly glycolic acid (PGA), are a novel strategy for cancer treatment, notably HNCs. Hydrophobic and hydrophilic medicines may be encapsulated in order to increase therapeutic efficacy and minimize side effects.

 There are several structural variants of PLGA, which are primarily determined by their molecular weights (Mw) and their molar ratios of lactic acid (LA) to glycolic acid (GA).^72^ The LA:GA ratio profoundly affects hydrophobicity, crystallinity, and degradation kinetics. For instance, high LA content increases hydrophobicity through LA methyl groups, slower water diffusion, and extends degradation time to months. Furthermore, higher Mw increases mechanical stability and reduces the initial burst release through denser matrices, while lower Mw accelerates drug degradation and release.^73^ Alternatively, a 50:50 ratio utilizes the hydrophilic properties of GA to promote rapid hydrolysis, resulting in rapid degradation, and burst release profiles that are optimized for acute therapeutic applications.^74^

 Further, the surface modification of PLGA enables the attachment of ligands or antibodies that target cancer cell receptors while protecting healthy tissues. ^75^ Hence, PLGA NPs are a promising cancer therapy due to their complex properties.^76^

 In vitro results show that particle size, surface charge, and encapsulation efficiency (EE) strongly influence absorption and efficacy. For instance, QbD-optimized PTX-PLGA NPs (53 nm, EE > 90%) reduced the IC50 in pharyngeal cancer cells by 50% compared to free PTX, due to increased internalization and sustained release.^77^ Multi-omics analysis of PTX-PLGA in FaDu cells revealed upregulated tubulin proteins and metabolites such as sphinganine, which promote apoptosis through synergistic effects of nanocarriers.^78^ Bi-drug systems, such as DOPA-coated GEM-PT-PLGA (Gemcitabin-Cisplatin), induced potent apoptosis in nasopharyngeal cells and sustained tumor inhibition in grafts, outperforming single-drug or free forms by improving compatibility and simultaneous delivery.^79^

 Moreover, stimulus-responsive designs enhance selectivity. Photothermal therapy and chemotherapy enabled EGFR-targeted PLGA/polydopamine-DOX NPs to achieve complete tumor removal without cardiotoxicity in vivo.^80^ Similarly, PLGA/Solutol HS15-DTX NPs improved solubility and release for various cancers, including HNC (Table 2).^81^

**Table 2:** PLGA/ PLGA copolymer NPs for HNC therapeutics

**Cancer Type**	**Polymer**	**Active Ingredient**	**Model Systems**	**Particle Characteristics**	**Dosing / Control Arms**	**Outcomes (IC**_50_ ** shift, tumor inhibition, etc.)**	**Short Analysis (Why some approaches performed better)**	**Ref.**
Head and neck (pharyngeal) carcinoma	PLGA nanoparticles (QbD-optimized)	Paclitaxel (PTX)	In vitro: human pharynx carcinoma cells	53 nm; ζ = −10.1 mV; EE > 90%; PDI = 0.221; spherical	Free PTX vs PTX–PLGA NPs	IC₅₀ reduced by ~50%; 10 × higher cumulative drug release over 72 h; 50% cell viability reduction vs 20% for free PTX	QbD-guided optimization produced ultra-small, high-loading PLGA NPs with improved cellular internalization and sustained release, leading to markedly higher cytotoxic efficacy.	^77^
Head and neck (hypopharyngeal) carcinoma	PLGA nanoparticles	Paclitaxel (PTX)	In vitro: FaDu human hypopharyngeal carcinoma cells	Not specified (PLGA-based NPs, PTX-loaded)	Free PTX vs blank PLGA NPs vs PTX–PLGA NPs	Higher apoptosis and cytotoxicity with PTX–PLGA NPs; altered expression of tubulin-associated and apoptosis-related proteins; increased metabolites (vitamin D, sphinganine, phosphatidylcholine)	Multi-omics revealed PTX–PLGA synergy enhancing apoptosis via proteomic and metabolic reprogramming; nanocarrier stress amplified PTX-induced cytoskeletal and metabolic disruption.	^78^
Nasopharyngeal carcinoma	PLGA nanoparticles (DOPA-coated dual-drug system)	Gemcitabine (GEM) + Cisplatin (PT)	In vitro: CNE2, SUNE1 cells; In vivo: nasopharyngeal xenograft mice	Nanosized; spherical; DOPA hydrophobic coating enhanced GEM encapsulation	Free drugs vs single-drug NPs vs dual-drug GEM–PT–PLGA NPs	Strong apoptosis induction; durable tumor growth inhibition; synergistic cytotoxicity > single or free drug treatments	DOPA coating improved dual-drug compatibility and encapsulation, enabling synergistic GEM–PT delivery; co-loaded PLGA NPs triggered amplified apoptosis and superior in vivo tumor suppression.	^79^
Head and neck, gastric, breast, and prostate cancers	PLGA/Solutol HS15 nanoparticles	Docetaxel (DTX)	In vitro (solid-state studies and release assays)	169 nm; negative ζ; spherical; amorphous DCT dispersed in PLGA	Controlled release at physiological pH	Enhanced DTX release rate; improved solubility and amorphization; stable nanosystem	Solutol HS15 improved emulsification and drug dispersion, producing controlled release and better solubility of hydrophobic docetaxel for local cancer therapy.	^81^
Head and neck carcinoma	PLGA/polydopamine (PD) core–shell nanoparticles (EGFR-targeted)	Doxorubicin (DOX)	In vitro: HNC cells; In vivo: xenograft mice	Core–shell; NIR-responsive; biodegradable; NP concentration–dependent self-regulated heating	Free DOX vs PLGA/PD–DOX ± NIR	Complete tumor ablation; no systemic toxicity; controlled drug release under NIR	Biodegradable PLGA/PD hybrid achieved EGFR-targeted thermochemotherapy; NIR-triggered PD shell enabled safe, selective heating and synergistic DOX release with minimal cardiotoxicity.	^80^

**Abbreviations:** PLGA: poly(lactic-co-glycolic acid), PTX: Paclitaxel, ζ: zeta potential, EE: encapsulation efficiency, PDI: polydispersity index, NPs: nanoparticles, IC₅₀: half maximal inhibitory concentration, FaDu: FaDu human hypopharyngeal carcinoma cell line, DOPA: 3,4-dihydroxyphenylalanine, GEM: Gemcitabine, PT: Cisplatin, CNE2: CNE2 nasopharyngeal carcinoma cell line, SUNE1: SUNE1 nasopharyngeal carcinoma cell line, DTX: Docetaxel, PD: polydopamine, EGFR: epidermal growth factor receptor, DOX: Doxorubicin, HNC: head and neck carcinoma, NIR: near-infrared

####  Chitosan and Chitosan Copolymers

 Chitosan NPs may be useful in challenging anatomical and physiological barriers of oral and upper gastrointestinal malignancies.

 In recent years, researchers have demonstrated the versatility of chitosan as a diagnostic, therapeutic, and prophylactic agent. Yang et al.^82^ revealed that folate-modified chitosan NPs significantly enhanced protoporphyrin IX accumulation and targeted delivery of 5-aminolevulinic acid (5-ALA) during PDT therapy. In OSCC models, Wang et al.^83^ demonstrated mitochondrial-targeted PDT and gene silencing using 5-ALA and shGBAS, resulting in significant cytotoxicity and tumor suppression in vitro and in vivo. The conjugates of chitosan amphiphilic oligosaccharide and arachidic acid have also been designed to generate self-assembly NPs with pH-sensitive DOX release, which perform better than free DOX in FaDu xenograft engraftment.^84^

 Moreover, there is a growing body of evidence that chitosan has potential use in gene therapy. As reported by Liu et al.^85^ and Zhuo et al.^86^ simple chitosan NPs effectively delivered siRNAs against C-erbB-2 in laryngeal cancer and TWIST in nasopharyngeal carcinoma, suppressing invasion and increasing radiosensitivity through ERK signaling, respectively. In esophageal squamous cell carcinoma, advanced designs such as carboxymethyl chitosan NPs loaded with DOX and siRNA-targeting MDR have reversed multidrug resistance through EGFR targeting and cytosolic escape.^87^ It has been demonstrated that radiation-induced E9-hTERT promoters driving dual suicide genes in 131I-labeled chitosan NPs provided excellent tumor specificity in dedifferentiated thyroid cancer. ^88^

 Moreover, Habibi et al.^89^ developed Janus NPs that combine chitosan’s cationic mucoadhesive properties with a PLGA controlled-release compartment to deliver tocilizumab. They successfully achieved deep penetration and potent chemoprevention in OSCC grafts, addressing a critical yet under-researched need for oral premalignant cancer field coverage prevention (Table 3). However, limitations, such as lack of comprehensive pharmacokinetic profiles, long-term toxicological evaluations, or quantitative comparison criteria, complicate clinical applications. Thus, chitosan-based nanoplatforms are considered pioneers in precision oncology, with an ability to overcome resistance and enable preventive strategies.

**Table 3:** Chitosan and its copolymer NPs for HNC therapeutics

**Cancer Type**	**Polymer**	**Active Ingredient**	**Model Systems**	**Particle Characteristics**	**Dosing / Control Arms**	**Outcomes (IC**_50_ ** shift, tumor inhibition, etc.)**	**Short Analysis (Why some approaches performed better)**	**Ref.**
Oral squamous cell carcinoma (OSCC)	Chitosan (CS) NPs	5-aminolevulinic acid (ALA) + shGBAS plasmid DNA	In vitro: WSU-HN6, CAL-27 cells; In vivo: oral cancer xenograft nude mice	Spherical; good dispersion/stability/hypotoxicity; ionic crosslinking (ALA) + electrostatic attraction (shGBAS); N/*P* ≥ 1/2	Not specified; CS-ALA-shGBAS vs controls (e.g., CS-ALA, CS-shGBAS)	Superior mitochondrial targeting; enhanced cell death via MTT/ROS assays; inhibited tumor growth in vivo; combined PDT-gene therapy synergy	Mitochondrial-targeted co-delivery of ALA (for PpIX-induced PDT) and shGBAS amplified ROS production and apoptosis, outperforming single therapies by addressing multifactorial OSCC resistance.	^83^
Oral cancer (head and neck subtype)	Chitosan-based nanoparticles (succinate-modified chitosan (SCHI) complexed with folic-acid-modified chitosan (fSCN))	5-aminolaevulinic acid (5-ALA)	In vitro: oral cancer cells	z-average diameter 110.0 nm; zeta potential 18.6 mV; stable in suspension; 72.8% drug loading efficiency; promoted lysosomal release via deprotonated SCHI	fSCN vs fSCNA (5-ALA-loaded); no significant differences in size/zeta post-loading	Enhanced engulfment via folate-receptor-mediated endocytosis; higher intracellular PpIX accumulation for photodynamic detection; improved drug release in lysosome	Folic-acid conjugation enabled targeted delivery to folate-receptor-overexpressing cancer cells, while succinate modification reduced chitosan-5-ALA attraction for lysosomal release, outperforming non-targeted systems in PpIX buildup for early detection.	^82^
Esophageal squamous cell carcinoma (ESCC)	Carboxymethyl chitosan modified with histidine, cholesterol, EGFR antibody (CHCE)	Adriamycin + MVP-siRNA + BCL2-siRNA	In vitro: KYSE510 and resistant 510K ESCC cells; In vivo: 510K xenograft nude mice	Spherical; size 90.26 nm (pH 7.4), 100.2 nm (pH 6.5), 129.1 nm (pH 5.4); low PDI; 85% siRNA encapsulation; serum stable 12 h	In vitro: PBS, CEA NPs, CEAM NPs, CEAB NPs, CEAMB NPs (siRNA 100 nM, Adriamycin 0.5 µg/mL, 48 h); In vivo: tail vein injection days 0,1,18; sacrifice day 39	MVP/BCL2 knockdown; reduced viability; G0/G1 arrest; 66.2% apoptosis; tumor volume reduction; increased caspase3	Multifunctional modifications enabled targeted co-delivery of chemo and gene therapy, enhancing intracellular efficacy against MDR mechanisms, leading to superior tumor inhibition compared to single-agent or non-targeted approaches.	^87^
Laryngeal cancer	Chitosan nanoparticles	C-erbB-2 siRNA	In vitro: Hep-2 cells	Approximately 100 nm	Not specified; treated with C-erbB-2-siRNA-chitosan NPs vs controls	Down-regulated C-erbB-2 expression; decreased cell invasion; induced apoptosis	Chitosan-mediated siRNA delivery effectively silenced C-erbB-2, reducing invasion and promoting apoptosis by targeting oncogenic pathways, superior to free siRNA due to enhanced cellular uptake and protection.	^85^
Nasopharyngeal carcinoma (NPC)	Chitosan nanoparticles	TWIST-siRNA	In vitro: CNE2 cells	Not specified; chitosan-encapsulated for siRNA delivery	Not specified; TWIST-CS/siRNA + irradiation vs controls (e.g., control-siRNA, no siRNA)	Suppressed TWIST; activated ERK (not JNK/p38); increased apoptosis/reduced viability post-irradiation	Efficient knockdown enhanced radiosensitivity via ERK-mediated EMT inhibition, outperforming controls in radioresistant cells.	^86^
Head and neck cancer (FaDu cells)	Chitosan oligosaccharide-arachidic acid (CSOAA) conjugate	Doxorubicin (DOX)	In vitro: FaDu human head and neck cancer cells; In vivo: FaDu tumor xenograft mouse model	Self-assembled; spherical; mean diameter 130 nm; positive zeta potential	Free DOX vs DOX-loaded CSOAA nanoparticles	Higher cellular uptake of DOX; sustained and pH-dependent release; negligible cytotoxicity of blank carrier; superior anti-tumor efficacy in vivo	Amphiphilic modification with arachidic acid enabled stable self-assembly, improved DOX solubilization, and pH-sensitive release in acidic tumor milieu, resulting in enhanced intracellular delivery and tumor inhibition compared to free drug.	^84^
Dedifferentiated thyroid carcinoma (deDTC)	Chitosan	Double suicide genes (TK/CD) driven by E9-hTERTp + ¹³¹I	In vitro: dFTC-133 cells, Nthy-ori3-1 cells	Nearly 80–120 nm; approximately round shaped with smooth surface	Vectors at 10 µg/ml; with/without prodrugs; controls: vector1 (¹³¹I + chitosan + E9-hTERTp-TK/CD), vector2 (chitosan + hTERTp-TK/CD), vector3 (¹³¹I + chitosan), vector4 (chitosan)	Enhanced CD/TK expression; decreased viability; increased apoptosis	The innovative 'gene switch' (radiation enhancer E9 + hTERTp) amplified suicide gene expression specifically in telomerase-active deDTC cells, while ¹³¹I provided dual therapeutic radiation, achieving superior specificity and potency over constitutive promoters.	^88^
Oral squamous cell carcinoma (OSCC)	Janus nanoparticles (JNP; cationic chitosan + hydrophobic PLGA compartments)	Tocilizumab (TCZ; IL-6 receptor antagonist)	In vitro: oral keratinocytes; ex vivo: full-thickness oral mucosal explants; In vivo: OSCC xenograft model	Monodispersed; average diameter 327 nm; PDI 0.245; high circularity > 0.90; mucoadhesive/mucopenetrating	TCZ-loaded JNP vs control groups	Controlled TCZ release; retained bioactivity (71% soluble IL-6Rα, 50% membrane-bound reduction); 76% breached stratum corneum, 41% reached basal layer; high xenograft inhibition, reduced proliferation/tumor size/ERG expression	Janus design synergized chitosan's mucoadhesion with PLGA's controlled release, enabling deep mucosal penetration and sustained IL-6R blockade for superior field-coverage chemoprevention over conventional systems.	^89^

**Abbreviations:** OSCC: Oral squamous cell carcinoma, CS: Chitosan, ALA: 5-aminolevulinic acid, shGBAS: short hairpin RNA targeting glioblastoma-amplified sequence, PDT: photodynamic therapy, N/P: Nanoparticle/Plasmid ratio, PpIX: protoporphyrin IX, MTT: 3-(4,5-dimethylthiazol-2-yl)-2,5-diphenyltetrazolium bromide, ROS: reactive oxygen species, SCHI: succinate-modified chitosan, fSCN: folic-acid-modified chitosan, mV: millivolt, ESCC: Esophageal squamous cell carcinoma, EGFR: epidermal growth factor receptor, CEAMB NPs: Carboxymethyl chitosan-based nanoparticles with Adriamycin MVP BCL2 siRNAs, DOX: Doxorubicin (Adriamycin), siRNAs: small interfering RNAs, MVP: major vault protein, BCL2: B-cell lymphoma-2, MDR: multidrug resistance, C-erbB-2: cellular erbB-2 (HER2), Hep-2: Hep-2 laryngeal cancer cell line, NPC: Nasopharyngeal carcinoma, TWIST: TWIST transcription factor, CNE2: CNE2 nasopharyngeal carcinoma cell line, EMT: epithelial-mesenchymal transition, FaDu: FaDu head and neck cancer cell line, CSOAA: Chitosan oligosaccharide-arachidic acid, deDTC: Dedifferentiated thyroid carcinoma, ¹³¹I: iodine-131, hTERTp: human telomerase reverse transcriptase promoter, JNP: Janus nanoparticles, PLGA: poly(lactic-co-glycolic acid), TCZ: Tocilizumab, PDI: polydispersity index, ERG: ETS-related gene

####  PEI/ PEI Copolymers

 The potential of polyethyleneimine (PEI) and its copolymers in cancer therapy has been highlighted. An investigation was conducted into the co-delivery of MDR1-siRNA and DOX using mesoporous silica NPs (MSNP) that were specially modified with PEI to minimize drug resistance in OSCC. The results revealed a 70% reduction in MDR1 gene expression and a 24.27% increase in apoptosis in KBV cells. Furthermore, an 81.64 % reduction in tumor size was observed during the 28-day in vivo treatment, indicating the enhanced therapeutic effects of PEI-modified NPs. These results suggest that employing PEI-modified NPs might be a potential technique for reducing drug resistance in OSCCs.^90^ Besides, Ma et al.^91^ [2017] highlighted enhancing therapeutic effects in OSCC by measuring the inhibition of the Wnt/β-catenin pathway by transfection of Wnt-1 siRNA with PEG-PEI-Ce6 NPs.

 Furthermore, the adverse effects associated with platinum-based medicines compelled Teng et al.^92^ to investigate the potential of developing NPs modified with PEI and PEG. They noticed that the NPs inhibited tumors efficiently, suggesting they may be employed for a wide range of applications, including cancer imaging and treatment in vivo and in vitro. Additionally, in the case of ESCC, a pH-sensitive nanosystem containing an innovative T7 peptide was combined with docetaxel and curcumin. The results indicate that the application of this nanosystem enhances cellular absorption, thereby producing a more potent anti-cancer effect.^93^ Furthermore, the targeted gene delivery of EGFR using GE11-PEG-PEI NPs demonstrated enhanced prognoses in cases of laryngeal cancer. By efficiently binding and condensing plasmid DNA, this nanocarrier attains a greater transfection efficiency in Hep-2 cells and nude mice (Table 4).^94^

**Table 4:** PEI/ PEI copolymer NPs for HNC therapeutics

**Cancer Type**	**Polymer**	**Active Ingredient**	**Model Systems**	**Particle Characteristics**	**Dosing / Control Arms**	**Outcomes (IC**_50_ ** shift, tumor inhibition, etc.)**	**Short Analysis (Why some approaches performed better)**	**Ref.**
Oral squamous cell carcinoma (OSCC; multidrug-resistant KBV cells)	Mesoporous silica nanoparticles (MSNP) modified with cationic polymer polyethylenimine (PEI)	Doxorubicin (DOX) + MDR1-siRNA	In vitro: KBV vincristine-resistant cells; In vivo: KBV xenograft BALB/c nude mice	Positively charged surface post-PEI modification; enabled simultaneous loading of DOX and MDR1-siRNA	Control vs DOX only vs MSNP-PEI-DOX/MDR1-siRNA	~70% MDR1 gene knockdown; 24.27% apoptosis induction at 48 h; 81.64% tumor volume reduction at 28 days in vivo (P < 0.05)	PEI surface modification conferred positive charge for efficient siRNA complexation and cellular uptake; co-delivery silenced P-gp efflux pump while releasing DOX intracellularly, synergistically overcoming MDR far beyond DOX monotherapy.	^90^
Oral squamous cell carcinoma (KB cells)	Polyethylene glycol-polyethyleneimine-chlorin e6 (PEG-PEI-Ce6) nanoparticles	Wnt-1 siRNA + Chlorin e6 (Ce6) photosensitizer	In vitro: KB oral squamous cell carcinoma cells	PEG-PEI-Ce6 NPs for efficient cytoplasmic siRNA delivery and PDT	PDT alone vs PEG-PEI-Ce6-mediated PDT vs PDT + Wnt-1 siRNA-loaded PEG-PEI-Ce6 NPs	Downregulated Wnt-1, β-catenin, vimentin; inhibited EMT; markedly enhanced cell growth inhibition and cancer cell killing post-PDT	Dual-functional PEG-PEI-Ce6 NPs enabled simultaneous photosensitization and efficient Wnt-1 siRNA delivery; EMT inhibition prevented PDT-induced tumor progression/recurrence, yielding superior cytotoxicity over PDT monotherapy.	^91^
Human laryngeal carcinoma (Hep-2 cells)	NaGdF4:Yb³⁺/Er³⁺ upconversion nanoparticles (UCNPs) modified with polyethyleneimine (PEI), poly(ethylene glycol) (PEG), and RGD peptide	Phenanthriplatin(IV) (Phen-Pt(IV)) prodrug	In vitro: Hep-2 cells; In vivo: Hep-2 xenograft mice; MR/UCL imaging	Water-dispersible; multifunctional (UCL green emission under 980 nm; T1-weighted MRI; RGD-targeted)	Not specified; Phen-Pt-conjugated UCNP@PEI–Phen-Pt–PEG–RGD vs controls	Obvious cytotoxicity (MTT); efficient cellular uptake (flow cytometry/ICP-MS); green UCL imaging; T1-MR contrast; high tumor inhibition rate in vivo	PEI enabled Phen-Pt(IV) conjugation and endosomal escape; PEG improved dispersibility; RGD conferred active targeting; multimodal imaging guided therapy while reductive activation released active Pt(II), outperforming non-targeted or non-conjugated analogs.	^92^
Esophageal cancer	Cyclodextrin-β-CD-PEI-PEG copolymer with T7 peptide modification (CM-β-CD-PEI-PEG-T7)	Docetaxel (DTX) + Curcumin (CUR)	In vitro: KYSE150, KYSE510 cells; In vivo: KYSE150 xenograft model	~100 nm; pH-responsive; T7-targeted; double emulsion synthesis; 10% DTX, 6.1% CUR loading	NP-DC vs T7-NP-DC vs docetaxel alone	Enhanced uptake with T7; pH-triggered release; superior growth suppression in xenograft vs non-targeted or single-drug	T7 peptide enabled transferrin receptor-mediated targeting; pH-sensitive release in tumor microenvironment; synergistic DTX-CUR combination outperformed non-targeted or monotherapy via improved bioavailability and controlled delivery.	^93^
Laryngeal cancer	Polyethylene glycol-polyethylenimine (PEG-PEI) with GE11 peptide	pORF-hTRAIL plasmid	In vitro: Hep-2 cells; In vivo: laryngeal xenograft nude mice	Small positively charged nanoparticles (< 200 nm inferred); dependent on N/P ratio	GE11-PEG-PEI/pORF-hTRAIL vs mPEG-PEI/pORF-hTRAIL vs saline	Greater transfection efficiency; significant tumor growth reduction (p < 0.05); increased apoptosis	GE11 conjugation enabled EGFR-targeted delivery, enhancing gene expression and therapeutic efficacy over non-targeted PEG-PEI in EGFR-overexpressing tumors.	^94^

Abbreviations: OSCC: Oral squamous cell carcinoma, KBV: multidrug-resistant KBV cells, MSNP: Mesoporous silica nanoparticles, PEI: polyethylenimine, DOX: Doxorubicin, MDR1-siRNA: MDR1-small interfering RNA, P-gp: P-glycoprotein, MDR: multidrug resistance, PEG-PEI-Ce6: Polyethylene glycol-polyethyleneimine-chlorin e6, Ce6: Chlorin e6, NPs: nanoparticles, PDT: photodynamic therapy, EMT: epithelial-mesenchymal transition, PEG: poly(ethylene glycol), RGD: arginine-glycine-aspartic peptide, Phen-Pt(IV): Phenanthriplatin(IV) prodrug, MR: magnetic resonance, UCL: upconversion luminescence, MRI: magnetic resonance imaging, ICP-MS: inductively coupled plasma mass spectrometry, Pt(II): platinum(II), β-CD: beta-cyclodextrin, CM-β-CD-PEI-PEG-T7: Carboxymethyl-beta-cyclodextrin-PEI-PEG-T7, DTX: Docetaxel, CUR: Curcumin, NP-DC: NP-docetaxel-curcumin, PAMAM: polyamidoamine dendrimer, G5: generation 5, B6: B6 peptide, TfR: transferrin receptor, GE11: GE11 peptide, EGFR: epidermal growth factor receptor, siRNA: small interfering RNA, eGFP-Luc: enhanced green fluorescent protein-luciferase

 Overall, PEI is a key player in the management of HNC due to its role in reversing drug resistance. Nevertheless, to ensure the safety of clinical applications, it is necessary to conduct detailed preclinical and mechanistic studies. As nanotechnology progresses, PEI hybrids may overcome these obstacles and provide precision in the face of HNC heterogeneity.

####  Other

 Regarding HNC, additional copolymers have been investigated. Yang & Qiu^95^ examined DTX-encapsulating MPEG-PLA-PAE NPs that are pH-sensitive and highlighted the benefits and limitations of large and small NPs. The researchers determined that poly (ethylene glycol)-poly(lactide)-poly (-amino ester) (DTX-pHPM) was the most efficacious growth inhibitor against oral epidermoid carcinoma. Furthermore, they emphasized the significance of particle size in determining their effectiveness. Moreover, methoxy poly (ethylene glycol)-b-polylactide (mPEG-PLA) copolymers represent a potential novel therapeutic approach for nasopharyngeal epidermal carcinoma in which an anti-angiogenic agent is combined with chemotherapy.^96^

## Conclusion

 Due to the complex treatment challenges presented by HNC with a high mortality rate, various treatments, including traditional treatments such as surgery, radiotherapy, and chemotherapy or using them in combination, have long existed. However, conventional agents suffer from poor tumor penetration, rapid systemic clearance, and non-specific toxicity, limiting dose intensity and long-term efficacy.​ This review emphasizes their unique properties and applications in cancer treatment by examining different types of PNPs and copolymers used in treating HNC. According to studies, PCL copolymers have played a fundamental and reassuring role in cancer treatment, especially in OSCC. These polymers with significant biodegradability facilitate dual drug delivery, intracellular absorption, and phototherapy. In addition, it increases apoptosis induction, tumor inhibition, and the ability to improve drug solubility, as seen in PCL-PEG micelles used for icaritin delivery. Also, the importance of PLGA-based NPs has been repeatedly mentioned in studies due to their capacity to deliver hydrophobic and hydrophilic drugs with controlled release. Also, the promising results of PTX-PLGA and GEM-PT NPs in nasopharyngeal and larynx cancer indicate the effectiveness of these NPs in the field of cancer.

 On the other hand, the high stability of chitosan has been used as an essential component in drug delivery in various fields, including increasing cellular uptake and targeting specific genes related to cancer, such as HNC. Preclinical biodistribution studies further support PNP utility, showing preferential tumor and lymph node accumulation, reduced off-target organ exposure, and mitigated toxicities like bone marrow suppression compared with free drugs. Early clinical signals from micellar taxanes/platinums (NCT06366945, NCT06301165) suggest feasibility in neoadjuvant settings, with potential for immunotherapy synergy.​ Nevertheless, translational hurdles persist. Heterogeneity in particle characterization, dosing, and models hampers comparability; long-term immunogenicity (e.g., anti-PEG antibodies), acidic degradation irritancy, and manufacturing scalability require rigorous evaluation. Meta-analyses confirm few mature trials, with high attrition rates due to immunosuppressive microenvironments and regulatory demands.​ Future research should focus on optimizing NP structures and reducing side effects using combination therapies. However, the potential of smart NPs in the targeted and effective treatment of HNC gives some promise for future cancer therapies.

 In summary, PNPs offer a promising platform to improve HNC therapy. They enable more precise drug delivery compared to conventional chemotherapy, with potential to enhance efficacy while reducing toxicity. Continued research is needed to address current limitations and achieve meaningful clinical benefits for patients.

 Edited: Head and neck cancers remain a significant global health challenge due to their complex etiology, aggressive behavior, and limited therapeutic options. Conventional treatment approaches—including surgery, radiotherapy, and chemotherapy—often produce substantial side effects and may fail to achieve satisfactory outcomes in advanced disease stages.

 Recent advances in nanotechnology have introduced innovative strategies for improving cancer diagnosis and treatment. Among these approaches, polymer-based nanoparticles have gained considerable attention because of their favorable physicochemical properties, including biocompatibility, biodegradability, and the ability to encapsulate a wide range of therapeutic agents.

 Polymeric nanoparticle systems offer several advantages over conventional drug delivery methods. These include enhanced drug stability, prolonged circulation time, controlled drug release, and improved tumor targeting through both passive and active mechanisms. Such properties enable higher drug concentrations at tumor sites while minimizing systemic toxicity.

 In head and neck cancer, polymer-based nanocarriers have demonstrated significant potential in delivering chemotherapeutic agents, genetic materials, and photosensitizers. Preclinical studies have reported improved therapeutic efficacy, reduced adverse effects, and enhanced tumor specificity compared with traditional treatments. Additionally, multifunctional nanoparticle systems capable of integrating therapeutic and diagnostic functions are opening new opportunities for personalized and precision medicine.

 However, despite encouraging preclinical results, the clinical translation of polymeric nanoparticle-based therapies remains limited. Challenges related to large-scale production, regulatory approval, long-term safety evaluation, and variability in tumor microenvironments must be addressed before these technologies can be widely adopted in clinical practice.

 Future research should focus on optimizing nanoparticle design, improving targeting strategies, and conducting well-designed clinical trials to evaluate safety and efficacy in patients with head and neck cancers. With continued interdisciplinary collaboration and technological innovation, polymer-based nanoparticle systems have the potential to significantly transform the therapeutic landscape of head and neck cancer in the coming years.

## Competing Interests

 The authors declare that they have no competing interests

## Ethical Approval

 This study was approved under the ethics code of IR.TBZMED.VCR.REC.1398.126.
